# Supplementary material for: A Systematic Review on Autism and Hyperserotonemia: State-of-the-Art, Limitations, and Future Directions
Source: Brain Sci. 2024 May 10;14(5):481. doi: 10.3390/brainsci14050481 (PMC11119126; doi:10.3390/brainsci14050481)
Supplement: Supplementary file 1 [file brainsci-14-00481-s001.zip › brainsci-2983173-supplementary.pdf]

Supplementary Material

Table S1. Adapted version of the Newcastle-Ottawa scale used in the present study for quality assessment (maximum 9 stars).

**Selection (maximum 4 stars):**
Is the case definition adeguate?<br>a) Yes, with standard diagnostic criteria (i.e., DSM, ICD, standardized diagnostic tools). *<br>b) Based on parental/self-reports or on clinical interviews.<br>c) No description.<br>***Representativeness of the cases:***<br>a) Consecutive or obviously representative series of cases. *<br>b) Potential for selection biases or not stated.<br>***Selection of controls:***<br>a) Community controls. *<br>b) Hospital controls or parents/siblings.<br>c) No description or absence of controls.<br>***Definition of controls:***<br>a) No history of disease (healthy controls). *<br>b) Other diagnoses.<br>c) No description or absence of controls.
**Comparability (maximum 2 stars):**
Comparability of cases and controls on the basis of the design or analysis<br>a) Study controls for age and sex. *<br>b) Study controls for IQ. *<br>c) Matching only for age or sex, or no matching neither for age and sex nor for IQ<br>d) No description or absence of controls
**Methods and procedure (maximum 3 stars):**
Experimental procedure:

a) The experimental procedure is well described and includes valid measures and methods. *

b) Poor description or no description of the procedure.

Same experimental procedure for cases and controls:

a) Yes. *

b) No.

c) No description or absence of controls

Drop-outs rate:

a) No drop-outs or same rate for all groups. *

b) Different rate or not defined

c) No description or absence of more than one group

Table S2. Details on quality assessment indices for the retrieved studies

Study	Selection				Comparability	Methods and procedure			Total
	Is the case definition adeguate?	Representativeness of the cases:	Selection of controls:	Definition of controls:	Comparability of cases and controls on the basis of the design or analysis	Experimental procedure:	Same experimental procedure for cases and controls:	Drop-outs rate	
Carpita et al., 2023	* (a)	(b)	* (a) and (b)	* (a)	(c)	* (a)	*(a)	* (a)	6
Cremone et al., 2023	* (a)	(b)	* (a) and (b)	* (a)	(c)	* (a)	*(a)	* (a)	6
Xiaoxue, 2023	* (a)	(b)	(b)	* (a)	* (a)	(b)	* (a)	* (a)	5
Zuniga-Kennedy et al., 2022	* (a)	* (a)	* (a)	*(a)	(c)	* (a)	*(a)	* (a)	7
Pagan et al., 2021	* (a)	(b)	* (a)	(c)	* (a)	* (a)	* (a)	(b)	5
Mostafa et al., 2021	* (a)	(b)	(b)	* (a)	* (a)	* (a)	* (a)	* (a)	6
Meyyazhagan et al., 2020	* (a)	(b)	(c)	(c)	* (a)	* (a)	* (a)	* (a)	5
Ali et al., 2020	(c)	(b)	(c)	(c)	(c)	(b)	* (a)	(b)	1
Javadfar et al., 2020	* (a)	* (a)	(c)	(c)	(d)	(b)	(c)	(c)	2
Chakraborti et al., 2020	* (a)	* (a)	* (a)	* (a)	(c)	* (a)	* (a)	* (a)	7
Wang et al., 2020	* (a)	(b)	* (a)	* (a)	* (a)	* (a)	*(a)	* (a)	7
Hua et al., 2020	* (a)	(b)	* (a)	* (a)	* (a)	* (a)	(b)	* (a)	6
Bridgemohan et al., 2019	* (a)	* (a)	(c)	(c)	(d)	* (a)	(c)	(c)	3
Wichers et al., 2019	* (a)	(b)	(c)	* (a)	** (a) and (b)	* (a)	* (a)	* (a)	7
Aaron et al., 2019	* (a)	* (a)	* (a)	* (a)	(c)	* (a)	* (a)	* (a)	7

Shuffrey et al., 2017	* (a)	* (a)	(c)	(c)	(d)	* (a)	(c)	(c)	3
Montgomery et al., 2018	* (a)	* (a)	(b)	* (a)	(c)	* (a)	* (a)	(b)	5
Lefevre et al., 2018	* (a)	* (a)	* (a)	* (a)	(c)	* (a)	* (a)	(b)	6
Abdulamir et al., 2018	* (a)	(b)	* (a)	* (a)	* (a)	* (a)	* (a)	(b)	6
Guo et al., 2018	* (a)	* (a)	* (a)	* (a)	* (a)	* (a)	* (a)	* (a)	8
Ormstad et al., 2018	* (a)	(b)	(b)	(b)	(c)	* (a)	* (a)	* (a)	4
Pagan et al., 2017	* (a)	(b)	* (a)	(c)	* (a)	* (a)	* (a)	(c)	5
Benabou et al., 2017	* (a)	(b)	* (a)	(c)	* (a)	*(a)	*(a)	(c)	5
Chen et al., 2017	* (a)	(b)	(b)	* (a)	(c)	* (a)	* (a)	(b)	4
Marler et al., 2016	* (a)	* (a)	(c)	(c)	(d)	* (a)	(c)	(c)	3
Chakraborti et al., 2016	* (a)	(b)	* (a)	* (a)	(c)	* (a)	* (a)	(b)	5
Kheirouri et al., 2016	* (a)	(b)	* (a)	* (a)	* (a)	* (a)	* (a)	* (a)	7
Francis et al., 2016	* (a)	* (a)	(c)	(c)	(d)	* (a)	(c)	(c)	3
Gebril et al，2015	* (a)	(b)	* (a)	* (a)	* (a)	* (a)	* (a)	* (a)	7
Chugani et al., 2016	* (a)	* (a)	(c)	(c)	(d)	* (a)	(c)	* (a)	4
Bijl et al., 2015	* (a)	* (a)	* (a) and (b)	* (a)	(c)	* (a)	* (a)	* (a)	7
Jaiswal et al., 2015	* (a)	* (a)	* (a) and (b)	* (a)	(c)	* (a)	* (a)	* (a)	7
Yang et al., 2015a	* (a)	(b)	* (a)	* (a)	* (a)	* (a)	* (a)	(b)	6
Yang et al., 2015b	* (a)	* (a)	* (a)	* (a)	* (a)	* (a)	* (a)	* (a)	8
Alabdali et al., 2014	* (a)	(b)	* (a)	* (a)	* (a)	* (a)	* (a)	* (a)	7

Kolevzon et al., 2014	* (a)	* (a)	(c)	(c)	(d)	* (a)	(c)	(c)	3
Gabriele et al., 2014	* (a)	* (a)	(b)	* (a)	(c)	* (a)	* (a)	* (a)	6
Pagan et al., 2014	* (a)	* (a)	* (a) and (b)	* (a)	* (a)	* (a)	* (a)	* (a)	8
Levin-Decanini et al., 2013	* (a)	* (a)	(b)	* (a)	(c)	* (a)	* (a)	(b)	5
Anderson et al., 2012	* (a)	(b)	* (a)	*(a)	* (a)	* (a)	* (a)	* (a)	7
Mostafa et al., 2011	* (a)	* (a)	* (a)	* (a)	* (a)	* (a)	* (a)	* (a)	8
El-Ansary et al., 2011	* (a)	* (a)	(b)	* (a)	(c)	* (a)	* (a)	* (a)	6
Sacco et al., 2010	* (a)	* (a)	(c)	(c)	(c)	* (a)	(c)	(c)	3
Kazek et al., 2010	(c)	* (a)	* (a)	* (a)	* (a)	* (a)	* (a)	* (a)	8
Kolevzon et al., 2010	* (a)	* (a)	(c)	(c)	(d)	* (a)	(c)	(c)	3
Mulder et al., 2009	* (a)	* (a)	(b)	(b)	** (a) and (b)	* (a)	* (a)	* (a)	7
Kemperman et al., 2008	* (a)	* (a)	(c)	(c)	(d)	* (a)	(c)	(c)	3
Warren and Singh, 2008	* (a)	* (a)	* (a)	* (a)	(c)	* (a)	* (a)	* (a)	7
Sacco et al., 2007a	* (a)	* (a)	* (a) and (b)	* (a)	(c)	* (a)	*(a)	(b)	6
Sacco et al., 2007b	* (a)	* (a)	(c)	(c)	(d)	* (a)	(c)	(c)	3
Coutinho et al., 2007	* (a)	* (a)	* (a)	* (a)	(c)	* (a)	* (a)	* (a)	7
Hranilovic et al., 2007	* (a)	* (a)	* (a)	* (a)	(c)	* (a)	* (a)	* (a)	7
Connors et al., 2006	* (a)	* (a)	* (a) and (b)	* (a)	(c)	* (a)	* (a)	* (a)	7
Weiss et al., 2006	* (a)	* (a)	* (a)	* (a)	(c)	(b)	* (a)	* (a)	6
Croonenberghs et al., 2005	* (a)	(b)	* (a)	* (a)	* (a)	* (a)	* (a)	* (a)	7

Spivak et al., 2004	* (a)	(b)	* (a)	* (a)	(c)	* (a)	* (a)	* (a)	6
Mulder et al., 2004	* (a)	* (a)	* (a) and (b)	* (a) and (b)	* (b)	* (a)	* (a)	* (a)	8
Coutinho et al., 2004	* (a)	* (a)	(b)	* (a)	(c)	* (a)	* (a)	(b)	5
Martin et al., 2003	* (a)	* (a)	(c)	(c)	(d)	* (a)	(c)	(c)	3
Vered et al., 2003	* (a)	(b)	* (a)	* (a)	* (a)	* (a)	* (a)	* (a)	7
Betancur et al., 2002	* (a)	* (a)	(b)	* (a) and (b)	(c)	(b)	* (a)	* (a)	5
Persico et al., 2002	* (a)	* (a)	(b)	* (a)	(c)	* (a)	* (a)	* (a)	6
Croonenberghs et al., 2000	* (a)	(b)	* (a)	* (a)	* (a)	* (a)	* (a)	* (a)	7
Leboyer et al., 1999	* (a)	* (a)	* (a) and (b)	* (a)	* (a)	* (a)	* (a)	* (a)	8
McBride et al., 1998	* (a)	* (a)	* (a)	* (a)	* (a)	* (a)	* (a)	* (a)	8
Singh et al., 1997	* (a)	(b)	* (a) and (b)	* (a) and (b)	** (a) and (b)	* (a)	* (a)	* (a)	8
Hérault et al., 1996	* (a)	* (a)	(c)	(c)	(d)	* (a)	(c)	(c)	3
Bouvard et al., 1995	* (a)	* (a)	(c)	(c)	(d)	* (a)	(c)	(c)	3
Torjman et al., 1995	* (a)	* (a)	* (a) and (b)	* (a) and (b)	** (a) and (b)	* (a)	* (a)	* (a)	9
Làszló et al., 1994	* (a)	(b)	* (a)	* (a)	(c)	(b)	* (a)	* (a)	5
Rolf et al., 1993	* (a)	* (a)	* (a)	* (a)	(c)	* (a)	* (a)	* (a)	7
Naffah-Mazzacoratti et al., 1993	* (a)	(b)	* (a)	* (a)	(c)	* (a)	* (a)	* (a)	6
Cuccaro et al., 1993	* (a)	* (a)	(b)	* (a)	(c)	* (a)	* (a)	* (a)	6
Leventhal et al., 1993	* (a)	* (a)	(c)	(c)	(d)	* (a)	(c)	(c)	3
Hérault et al., 1993	* (a)	* (a)	* (a)	* (a)	(c)	* (a)	* (a)	* (a)	7

Yuwiler et al., 1992	* (a)	(b)	* (a) and (b)	* (a) and (b)	(c)	* (a)	* (a)	* (a)	6
Duker et al., 1991	* (a)	(b)	(c)	(c)	(d)	* (a)	(c)	(c)	2
Perry et al., 1991	* (a)	* (a)	* (a) and (b)	* (a)	* (a)	* (a)	* (a)	* (a)	8
Piven et al., 1991	* (a)	* (a)	* (a)	* (a)	(c)	* (a)	* (a)	* (a)	7
Stern et al., 1990	* (a)	* (a)	(c)	(c)	(d)	* (a)	(c)	(c)	3
Cook et al., 1990	* (a)	* (a)	(b)	* (a)	* (b)	* (a)	* (a)	* (a)	7
Oades et al., 1990	* (a)	(b)	(c)	(c)	(d)	* (a)	(c)	(c)	2
Leventhal et al., 1990	* (a)	* (a)	(b)	* (a)	(c)	* (a)	* (a)	* (a)	6
Abramson et al., 1989	* (a)	* (a)	* (a)	* (a)	* (a)	* (a)	* (a)	* (a)	8
Ekman et al., 1989	* (a)	* (a)	(c)	(c)	(d)	*(a)	(c)	(c)	3
McBride et al., 1989	* (a)	* (a)	* (a)	* (a)	* (a)	* (a)	* (a)	* (a)	8
Sherman et al., 1989	* (a)	(b)	(c)	(c)	(d)	* (a)	(c)	(c)	2
Minderaa et al., 1989	* (a)	* (a)	* (a)	* (a)	* (a)	* (a)	* (a)	* (a)	8
Coggins et al., 1988	(b)	(b)	(c)	(c)	(d)	*(a)	(c)	(c)	1
Cook et al., 1988	(c)	(b)	(b)	* (a)	(c)	* (a)	* (a)	* (a)	4
Geller et al., 1988	* (a)	* (a)	* (a) and (b)	* (a) and (b)	* (a)	* (a)	* (a)	* (a)	8
Launay et al., 1988	* (a)	* (a)	(b)	* (a)	* (a)	* (a)	* (a)	* (a)	7
Kuperman et al., 1987	* (a)	* (a)	(c)	(c)	(d)	* (a)	(c)	(c)	3
Badcock et al., 1987	* (a)	* (a)	* (a) and (b)	* (a) and (b)	(c)	* (a)	* (a)	* (a)	7
Launay et al., 1987	* (a)	* (a)	(b)	* (a)	* (a)	* (a)	* (a)	* (a)	7

Minderaa et al., 1987	* (a)	* (a)	* (a)	* (a)	* (a)	* (a)	* (a)	* (a)	8
Anderson et al., 1987	* (a)	* (a)	* (a)	* (a)	(c)	* (a)	* (a)	* (a)	7
Ho et al., 1986	* (a)	(b)	* (a) and (b)	* (a) and (b)	(c)	* (a)	* (a)	* (a)	6
Stubbs et al., 1986	* (a)	(b)	(c)	(c)	(d)	(b)	(c)	(c)	1
Israngkun et al., 1986	* (a)	* (a)	* (a)	* (a)	* (a)	* (a)	* (a)	* (a)	8
Piggott et al., 1986	* (a)	(b)	(c)	(c)	(d)	(b)	(c)	(c)	1
August et al., 1985	(c)	(b)	(c)	(c)	(d)	* (a)	(c)	(c)	1
Kuperman et al., 1985	* (a)	* (a)	(b)	* (a)	(c)	* (a)	* (a)	* (a)	6
Hoshino et al., 1984	* (a)	* (a)	* (a)	* (a)	(c)	(b)	* (a)	* (a)	6
August et al., 1984	* (a)	* (a)	* (a)	* (a)	* (a)	(b)	* (a)	* (a)	7
Ritvo et al., 1984	* (a)	* (a)	(c)	(c)	(d)	* (a)	(c)	(c)	3
Ritvo et al., 1983	* (a)	* (a)	(c)	(c)	(d)	* (a)	(c)	(c)	3
Rotman et al., 1980	(c)	(b)	(c)	(c)	(d)	* (a)	(c)	(c)	1
Hanley et al., 1977	* (a)	(b)	* (a) and (b)	* (a) and (b)	(c)	* (a)	*(a)	* (a)	6
Takahashi et al., 1977	(b)	* (a)	* (a) and (b)	* (a) and (b)	* (a)	* (a)	* (a)	* (a)	7
Takahashi et al., 1976	* (a)	* (a)	* (a) and (b)	* (a) and (b)	* (a)	* (a)	* (a)	* (a)	8
Yuwiler et al., 1975	(b)	* (a)	* (a) and (b)	* (a) and (b)	(c)	* (a)	* (a)	* (a)	6
Ritvo et al., 1971	(b)	(b)	(c)	* (a)	(c)	* (a)	* (a)	* (a)	4
Yuwiler et al., 1971	(b)	(b)	(b)	(b)	(c)	* (a)	* (a)	* (a)	3
Ritvo et al., 1970	(b)	* (a)	* (a) and (b)	* (a)	(c)	* (a)	* (a)	* (a)	6

***Note:* the specific fulfilled criterion for the assignment or non-assignment of each star is specified in brackets.**									

Table S3. Peripheral serotonin concentration: matrix-specific data.

**Matrices**	**Detailed results of literature review**
Whole Blood	Whole Blood was the matrix used in 61% of cases (71 studies). Of these, 79% focused on absolute concentration, 6% on the ratio between the 5-HT and the number of platelets, and 9% on both. Six papers did not report this information. Out of 60 studies that measured the 5-HT concentration in whole blood, (36) 60% reported hyperserotonemia in people with ASD compared to healthy controls. Only 2 showed hyposerotonemia, 9 showed no differences, and 13 lacked unaffected controls or reference values. Different results were obtained by the studies focusing on the ratio between 5-HT concentration and platelet counts. Six reported hyperserotonemia in the ASD patients, 3 no differences between ASD patients and the control group, while in 1 paper, this result was not shown.
Platelet Rich Plasma (PRP)	Eighteen studies analysed 5-HT in PRP. They all used centrifugation to separate the platelet and plasma from the other blood components. There are similarities and differences between the parameters involved in PRP preparation procedures. The used range of Relative Centrifugal Field (RCF expressed in g) was from 80 to 1050 g. In 4 studies, the centrifugation was repeated two times. Different timing of centrifugation was used. The range was from 2 to 30 minutes. Four studies reported that the temperature was controlled at 4°C. Six studies measured 5-HT absolute concentration, 10 ratios between 5-HT concentration and platelet count (also expressed as ratio between ng of serotonin and mg of protein), and 3 both. In 1 study, the result was not reported. Ten out of 13 studies that considered 5-HT/platelets ratio or both ratio and absolute 5-HT concentration showed hyperserotonemia in the ASD population, 1 paper showed hyposerotonemia, 1 paper showed no alteration and 1 did not report this evaluation. Six out of 9 studies that expressed the results as absolute concentration detected hyperserotonemia inside the patients' population, 2 showed no differences, and 1 did not report this evaluation. On the whole, 72% of the studies reported hyperserotonemia.
Platelet Poor Plasma (PPP) or Plasma	Eighteen papers analysed PPP. Also, in this case, different procedures were used to prepare PPP, all based on centrifugation to separate PPP from the cells. The Relative Centrifugal Field (RCF expressed in g) ranged from 1000 to 12.000 g. In only one study, the centrifugation was performed 2 times for each sample. Different timing of centrifugation was used, ranging from 4 to 30 minutes. No centrifugation temperature was specified. Three papers did not mention the method to obtain the plasma. In contrast to the other matrix, in which a predominance of hyperserotonemia in the ASD population was detected, it was found only in 7 out of 18 PPP hyperserotonemia. Furthermore, hyposerotonemia was found in 6 out of 18 PPP, no difference in 3, and not reported in 2.
Serum	Serum was analysed in 13 articles. Of these, 9 studies detected hyperserotonemia in ASD patients, and 2 did not. Two of them were clinical trials with no reference population.
